# Fabrication of a Flexible PDMS/MWCNTs Composite Sensor Based on a Triboelectric Nanogenerator (Teng) and Its Application in Pulse Monitoring

**DOI:** 10.3390/polym18182291

**Published:** 2026-09-19

**Authors:** Longfei Zhang, Guowei Gao

**Affiliations:** 1 Beijing Key Laboratory of Sensor, Beijing Information Science and Technology University, Beijing 100101, China; 2 Key Laboratory of Modern Measurement and Control Technology, Ministry of Education, Beijing Information Science and Technology University, Beijing 100192, China

**Keywords:** sacrificial template method, porous structure, flexible triboelectric nanogenerator sensor, PDMS/MWCNTs composite film, pulse detection

## Abstract

In view of the demand for wearable physiological monitoring, porous PDMS/MWCNTs composite films were fabricated via the sacrificial template method in this work. Output performance tests were carried out on films with different sacrificial particle sizes, film thicknesses, and multi-walled carbon nanotube (MWCNT) mass fractions. Scanning electron microscopy (SEM) was employed for structural morphology characterization. The optimal particle size, thickness, and doping ratio were confirmed to be 63 μm, 3 mm, and 9 wt%, respectively, and flexible sensor arrays based on triboelectric nanogenerators were further fabricated. In this paper, a complete signal conditioning system consisting of a flexible sensor array and a signal acquisition circuit (including a power supply unit, reset circuit, signal conditioning module, and core control chip) was designed to realize data transmission and upper computer display. Pulse signals from four volunteers were collected in five groups, respectively, for comparative analysis. Experimental results reveal that multiple sets of pulse data from the same subject show high consistency. Slight deviations within 0.38 V are merely caused by breathing and other subtle interference factors. These results verify that the prepared sensor possesses favorable sensitivity and repeatability.

## 1. Introduction

With the accelerating pace of life, public attention to health has significantly increased. Due to mounting life pressures and escalating food safety challenges, the risk of cardiovascular diseases, including coronary heart disease [1] and hypertension [2], has risen substantially, posing severe threats to human health. Consequently, monitoring human health status has become of paramount importance. Currently, a major focus of medical research lies in the detection and analysis of human vital signs, encompassing key parameters such as blood pressure [3], pulse [4], heart rate [5], and respiration [6]. In particular, pulse monitoring remains a critical diagnostic approach, where physicians assess patients’ health conditions by palpating the pulse at the radial artery to perceive its strength and rhythm. Especially with the advancement of wearable technology and flexible sensors, the effective integration of traditional Chinese medicine diagnostic techniques with modern medicine has brought innovative developments to conventional pulse diagnosis methods. In particular, the emergence of triboelectric nanogenerator (TENG) technology has introduced novel detection approaches for pulse diagnosis, which enable self-powered sensing, quantitative pulse signal collection, and digitalized monitoring to facilitate the modern development of traditional Chinese medicine pulse diagnosis.

In 2017, Fan and his team [7] successfully fabricated porous materials containing multi-walled carbon nanotubes (CNT) for TENG devices using NaCl particles as templates; meanwhile, Yu and his team [8] employed citric acid monohydrate (CAM) particles as templates to produce porous PDMS sponges applicable for oil–water separation. In 2019, the triboelectric sensor reported in a relevant study possessed a cycling durability of 2000 cycles and enabled long-term stable operation under self-powered conditions [9]. In 2021, the MXene/cotton-fabric-based piezoresistive sensor delivered a cycling durability of more than 1000 cycles and was capable of acquiring various human physiological signals [10]. In 2021, HE Y and colleagues [11] proposed a TENG-based self-powered pressure sensor with ultra-high sensitivity utilizing microsphere structures within polydimethylsiloxane (PDMS) for pulse detection applications. In 2022, the research team [12] fabricated a flexible capacitive tactile sensor via electrospinning, and the device exhibited performance degradation after 1000 cycles. In 2022, Yu Sun [13] et al. designed a closed-loop self-powered low-level vagus nerve stimulation (LL-VNS) system based on hybrid nanogenerators (H-NG), capable of real-time monitoring of patients’ pulse wave status. In the same year, Puchuan Tan [14] et al. developed an artificial intelligence-enhanced blood pressure monitoring wristband sensor (BPPW) based on nanogenerators. In 2022, Chen et al. [15]. successfully fabricated a 3D double-sided interlocked fabric triboelectric nanogenerator (3DFIFTENG) by weaving cotton yarn and nylon 66 yarn. Meanwhile, Lou’s group [16] reported a triboelectric-sensing textile fabricated by weaving stainless steel wires wrapped with nylon filaments and polytetrafluoroethylene (PTFE) filaments, which acted as the positive and negative electrode layers, respectively. In 2023, Zhang et al. [17]. fabricated a superhydrophobic poly(vinylidene fluoride) nanofibrous membrane via electrospinning, based on which an all-fibrous superhydrophobic triboelectric nanogenerator (FTENG) was designed. Jiang’s group [18] combined MXene nanosheets with polyvinyl alcohol (PVA) and constructed a flexible, fully electrospun triboelectric nanogenerator. In recent years, textile and flexible triboelectric nanogenerators (TENGs) have been rapidly developed for wearable physiological and motion monitoring. In 2023, Chen et al. [19] developed an improved flexible triboelectric nanogenerator (TENG) based on MXene/poly(methyl methacrylate) (PMMA) composite electrodes featuring tile-like nanostructures (MP-TENG), demonstrating great potential for wireless human health monitoring. Similarly, Yu et al. [20] reported a flexible and stretchable single-electrode cotton textile TENG in the same year, which adopted breathable cotton–spandex blended fabric (95% cotton and 5% spandex) as the flexible electrode. This device maintained a stable output voltage of approximately 0.3 V under an applied force of 4 N and a frequency of 2.5 Hz, enabling effective detection of human motion behaviors. Further advancing wearable TENG technology, Shi et al. [21] fabricated a fabric-based integrated TENG (F-TENG) in 2025 by utilizing waterborne polyurethane (WPU) as both the waterproof encapsulation layer and friction layer. The optimized F-TENG could produce reliable output voltages for carotid pulse and respiratory signal acquisition, exhibiting excellent capability to capture subtle physiological characteristics.

In summary, the fabrication processes of most sensors remain complex and costly, limiting the large-scale production of TENGs [22]. This paper employs sugar granules as sacrificial templates. Compared with widely used inorganic salt and CAM templates reported in previous studies, sugar particles can be readily removed by mild water immersion without toxic solvents or rigorous etching treatments, which endows this strategy with relatively simple operation and low material cost. Most existing studies on porous TENGs only optimize single structural parameters (e.g., pore size and conductive filler loading) independently. Unlike these conventional strategies, this work proposes a facile sugar sacrificial template method to synchronously construct porous architectures. PDMS with high electronegativity is chosen as the device matrix, and sugar granules are utilized as sacrificial templates to prepare porous composite films with outstanding torsion and compression resistance, which greatly improves the mechanical flexibility of the material. The excellent electron-trapping ability of PDMS enables efficient charge transfer at the friction interface. Moreover, the incorporation of multi-walled carbon nanotubes (MWCNTs) optimizes the dielectric properties of the composite film, further boosting the electrical output performance of the triboelectric sensor. Pore size, film thickness, and MWCNT doping content are three core parameters dominating the comprehensive performance of the sensor. The pore size directly affects the flexibility, air permeability, and electrical output of the composite film; film thickness regulates the wearing comfort and triboelectric output in practical tests; and MWCNT doping serves as an effective means to modulate the overall output performance of the device. This integrated fabrication route eliminates complicated multi-step etching processes and toxic template removal treatments. The resulting flexible sensor realizes non-invasive acquisition of human pulse signals, offering a reliable and low-cost alternative for wearable physiological monitoring devices. To satisfy the requirements of practical wearable scenarios, the developed porous PDMS/MWCNT sensor adopts a composite film as the outer encapsulation layer. Double-sided Kapton adhesive tape is used to firmly bond the functional composite film with copper electrodes on flexible printed circuit (FPCB) substrates. The encapsulated device achieves stable conformal contact with human skin during pulse detection. This packaging design avoids bulky rigid structures and completely retains the inherent flexibility of the sensor. In future studies, further optimization of encapsulation materials and structural configurations will be conducted to improve the environmental anti-interference ability of the device and realize long-term stable on-body physiological monitoring.

## 2. Materials and Methods

### 2.1. Materials and Instruments

#### 2.1.1. Materials

PDMS liquid prepolymer and curing agent (Sylgard 184 kit, Dow Corning, Midland, MI, USA), MWCNTs (purity of 99%, outer diameter of 8–15 nm, length of 8–14 μm, and specific surface area ≥250 m^2^ g^−1^. Shenzhen Suiheng Carbon Nanotube Technology Co., Ltd., Shenzhen, China), sugar granules (Guangxi Yisheng Sugar Industry Co., Ltd., Guangxi, China), deionized water ((Grade I) Sinopharm Chemical Reagent Co., Ltd., Shanghai, China), acrylic mold (20 mm (width) × 60 mm (length), with thicknesses of 1.0 mm, 3.0 mm, and 5.0 mm. Senya Plastic Plate Material, Hebei, China), mortar and pestle (Anserui Experimental Equipment Co., Ltd., Hefei, China), and mesh sieve (Shaoxing Jinhang Instrument Co., Ltd., Zhejiang, China).

#### 2.1.2. Equipment

Analytical balance (ME204E, Mettle-Toledo Instruments Co., Ltd., Zurich, Switzerland), pipette (Shanghai Hen qi Instrumentation Co., Ltd., Shanghai, China), water bath constant-temperature heating magnetic stirrer (DF-101S, Shanghai Li Chen bang xi Instrument Technology Co., Ltd., Shanghai, China), constant-temperature drying oven (101-0A, Shanghai Kantian Experimental Instrument Co., Ltd., Shanghai, China), optical microscope (Primostar3, Carl Zeiss AG, Jena, Germany), Nova Field Emission Scanning Electron Microscope (Nova Nano SEM 450, Shanghai Jiu Xin Instrumentation Co., Ltd., Shanghai, China), FT2000 Flexible Electronics Tester (MF-FT2000, Prtronic, Shanghai MI fang Electronic Technology Co., Ltd., Shanghai, China), beaker, graduated cylinder (Shuniu Glassware Co., Ltd., Chendu, China), and VAC-V2 gas permeability tester (Labthink Instruments Co., Ltd., Jinan, China).

### 2.2. Fabrication of Triboelectric Nanogenerator (TENG) Films

First, a certain amount of PDMS elastomer was weighed and subjected to vacuum treatment. Subsequently, MWCNTs were incorporated into the PDMS elastomer at MWCNT-to-PDMS mass ratios of 7 wt%, 9 wt%, and 11 wt%. The mixture was stirred at a rotating speed of 500 r/min for 20 min. Thereafter, sugar particles were added to the MWCNT/PDMS mixture, and the mixture was continuously stirred at the same rotating speed for 30 min at room temperature to achieve uniform dispersion.

**Figure 1:**
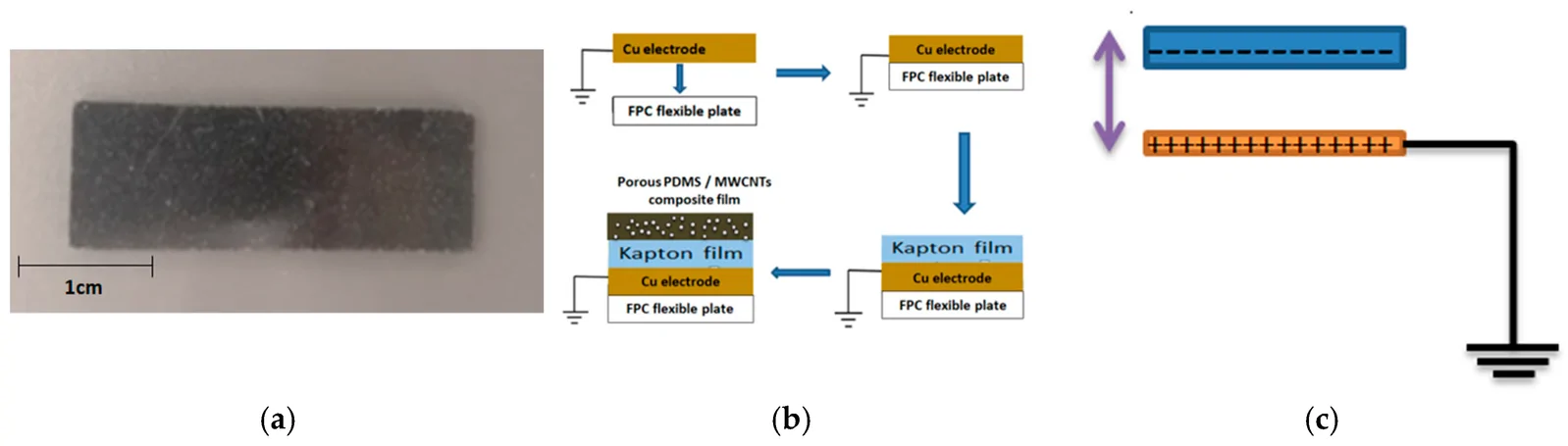
(**a**). Porous structure doped with MWCNTs; (**b**). Assembly process of single-electrode porous PDMS/MWCNTs composite TENG; (**c**). Schematic working mechanism of the single-electrode TENG.

A certain amount of curing agent was then added according to the mass ratio of PDMS elastomer to curing agent of 10:1, followed by another 30 min of stirring to ensure homogeneous mixing. A 30 min vacuum degassing process was conducted to eliminate air bubbles formed during stirring. The obtained mixture was poured into three molds of different sizes and then placed in a constant-temperature oven at 85 °C for 2 h to complete thermal curing and film formation. The cured films were immersed in deionized water and heated at 85 °C for 1 h. To completely remove the embedded sugar particles, the water was replaced every 20 min to eliminate residual sugar templates. After template removal, the wetted films were transferred to an oven and dried at 85 °C for 1 h to eliminate residual internal moisture. Sample mass was monitored periodically throughout drying. The constant sample mass observed after 1 h confirmed the complete removal of residual water under these conditions. Finally, porous PDMS/MWCNTs composite films were successfully fabricated, and the as-prepared films are presented in Figure 1a. The prepared porous PDMS/MWCNTs composite films were cut to a size of 1 × 1 cm to match the contact area of fingers for pulse diagnosis. The porous PDMS/MWCNTs composite film served as the main friction layer of the TENG. As shown in Figure 1b, double-sided Kapton tape, which prevents the leakage of transferred charges, was adopted to closely bond the composite film to the copper electrode of the flexible printed circuit board (FPCB) to achieve gap-free assembly. On this basis, a single-electrode porous composite TENG was fabricated. In this single-electrode TENG configuration, charge transfer occurs between the porous composite film and the external ground electrode. When external pressure drives contact–separation motion between the dielectric film and the contact object, electrostatic charges are generated on the dielectric surface. The potential difference induced by surface charge variation drives electron exchange between the electrode and ground, generating measurable open-circuit voltage signals, as shown in Figure 1c.

### 2.3. Design of Pulse Signal Data Acquisition

**Figure 2:**
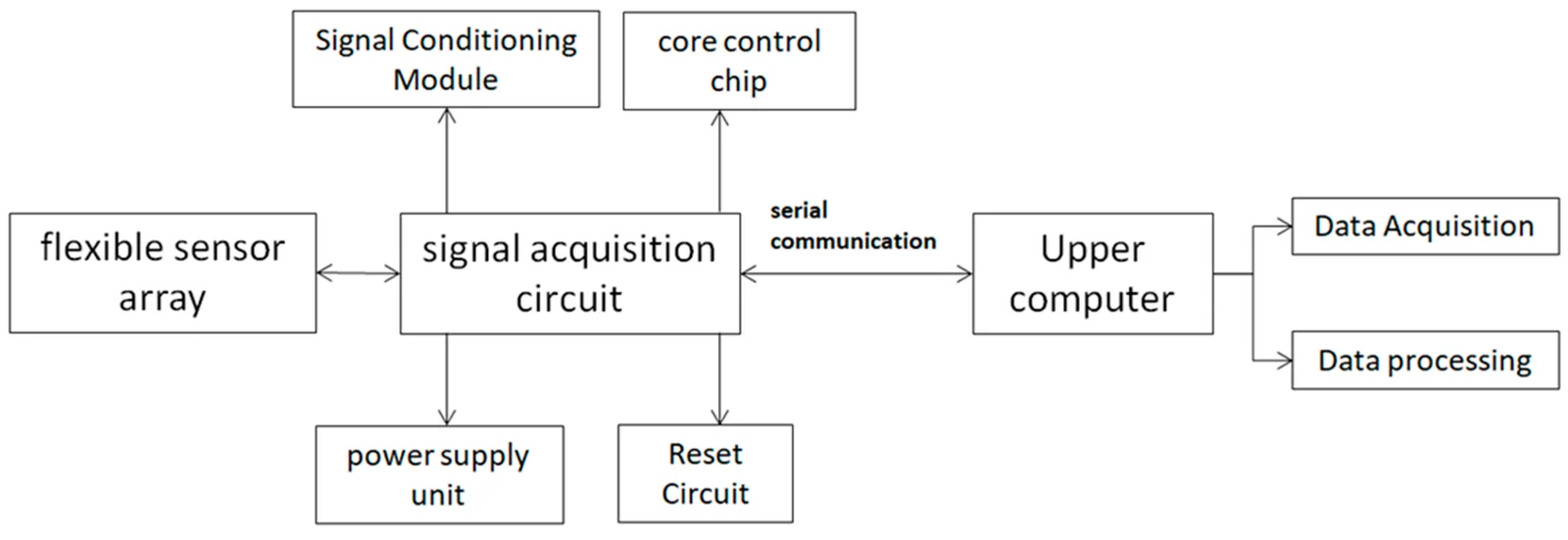
Frame diagram of flexible sensor signal acquisition system.

Signal conditioning was implemented by a dedicated signal acquisition circuit. A 1 × 3 single-electrode sensor array was adopted as the signal sensing front-end. Serial data transmission between the system and the upper computer was realized based on the Universal Synchronous/Asynchronous Receiver/Transmitter (USART) protocol, enabling real-time visual monitoring of the acquired data on the upper computer interface. The overall architecture of the signal acquisition system is illustrated in Figure 2 and consists of four functional modules: a flexible sensor array, a signal acquisition circuit (including a power supply unit, reset circuit, signal conditioning module, and core control chip), serial communication, and an upper computer. When single or multiple sensing units in the array are subjected to external pressure loading, surface charges are generated on the electrodes owing to the charge induction effect of the internal composite films. The analog signals are first processed by the signal conditioning module for amplification, followed by anti-interference and decoupling filtering via capacitors. Afterward, the conditioned signals are transmitted to the microcontroller for analog-to-digital conversion (ADC). The converted digital signals are uploaded to the upper computer through the serial communication interface. The upper computer conducts further data processing to realize pulse signal acquisition and real-time waveform visualization.

## 3. Results and Discussion

### 3.1. Performance Characterization of Porous PDMS/MWCNTs Composite Films

#### 3.1.1. Effect of Pore Size on Output Performance

**Figure 3:**
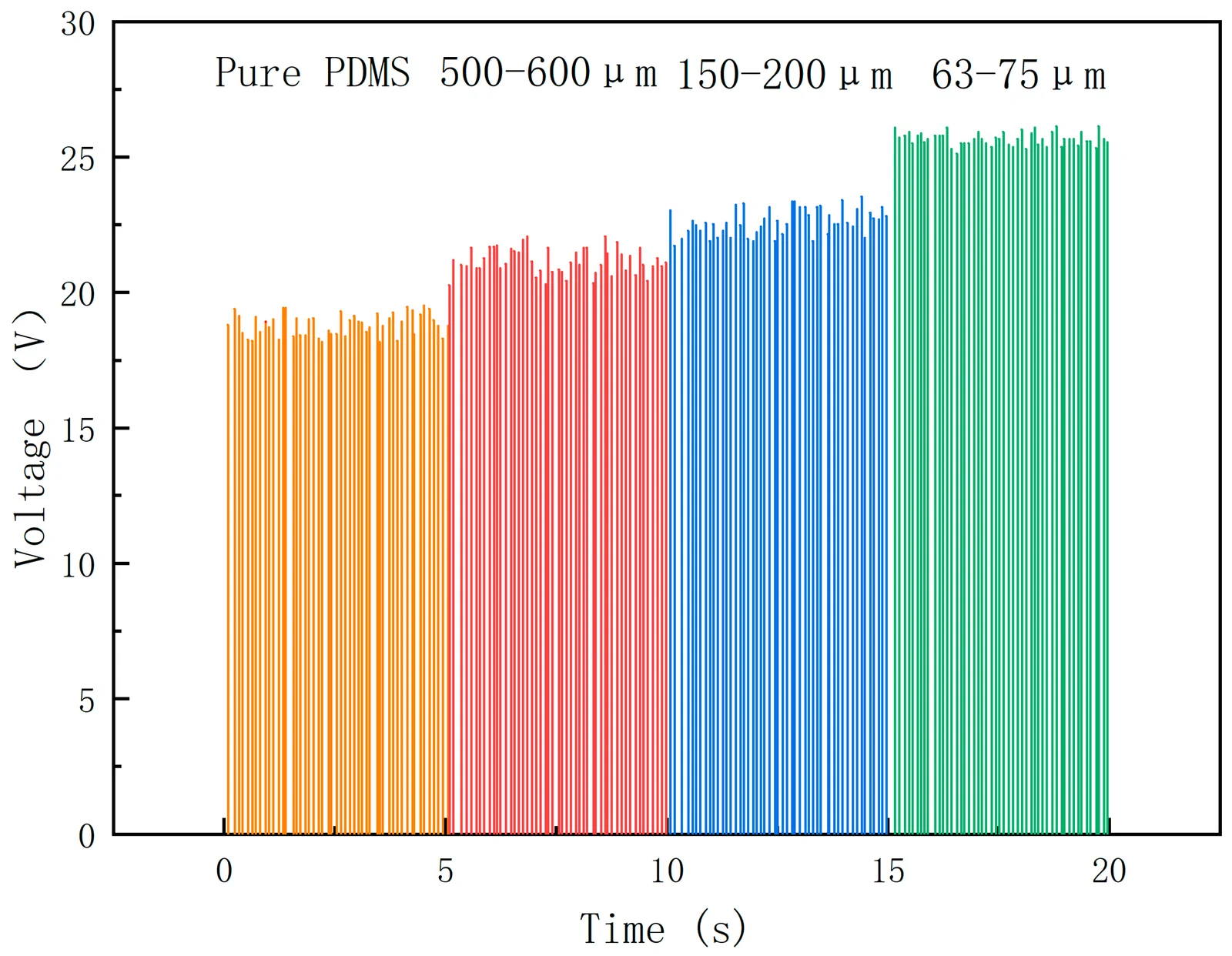
Correlation between pore size and output performance.

To investigate the relationship between pore size and output performance, four undoped films (one control sample and three samples with varied pore sizes) were selected as test samples, all with a uniform thickness of 3 mm. Using a flexible electronic test system (MF-FT2000, Prtronic, Shanghai MI fang Electronic Technology Co., Ltd., Shanghai, China) with an acrylic plate serving as the moving layer, the device was loaded with a periodic applied force of 80 N at 1 Hz under a square-wave waveform via a closed-loop force control mode. The test results are the peak values of the open-circuit voltage shown in Figure 3. The curves are horizontally offset by 2.5 s for better visual clarity. It can be concluded from the analysis that the output voltage increases gradually with the reduction in internal pore size. This phenomenon confirms that under equal sugar loading, smaller sacrificial templates provide more pore-forming units. Consequently, the number of internal pores inside the film increases, which further enlarges the internal friction area of the film. Meanwhile, compared with pure PDMS, the films with pore sizes of 500 μm and 150 μm exhibit only a slight improvement in output performance. In contrast, the film with a pore size of 63 μm achieves a much more remarkable performance enhancement. Therefore, the 63 μm pore size with optimal output performance is selected as the fundamental condition for subsequent tests.

#### 3.1.2. Relationship Between Film Thickness and Output Performance

**Figure 4:**
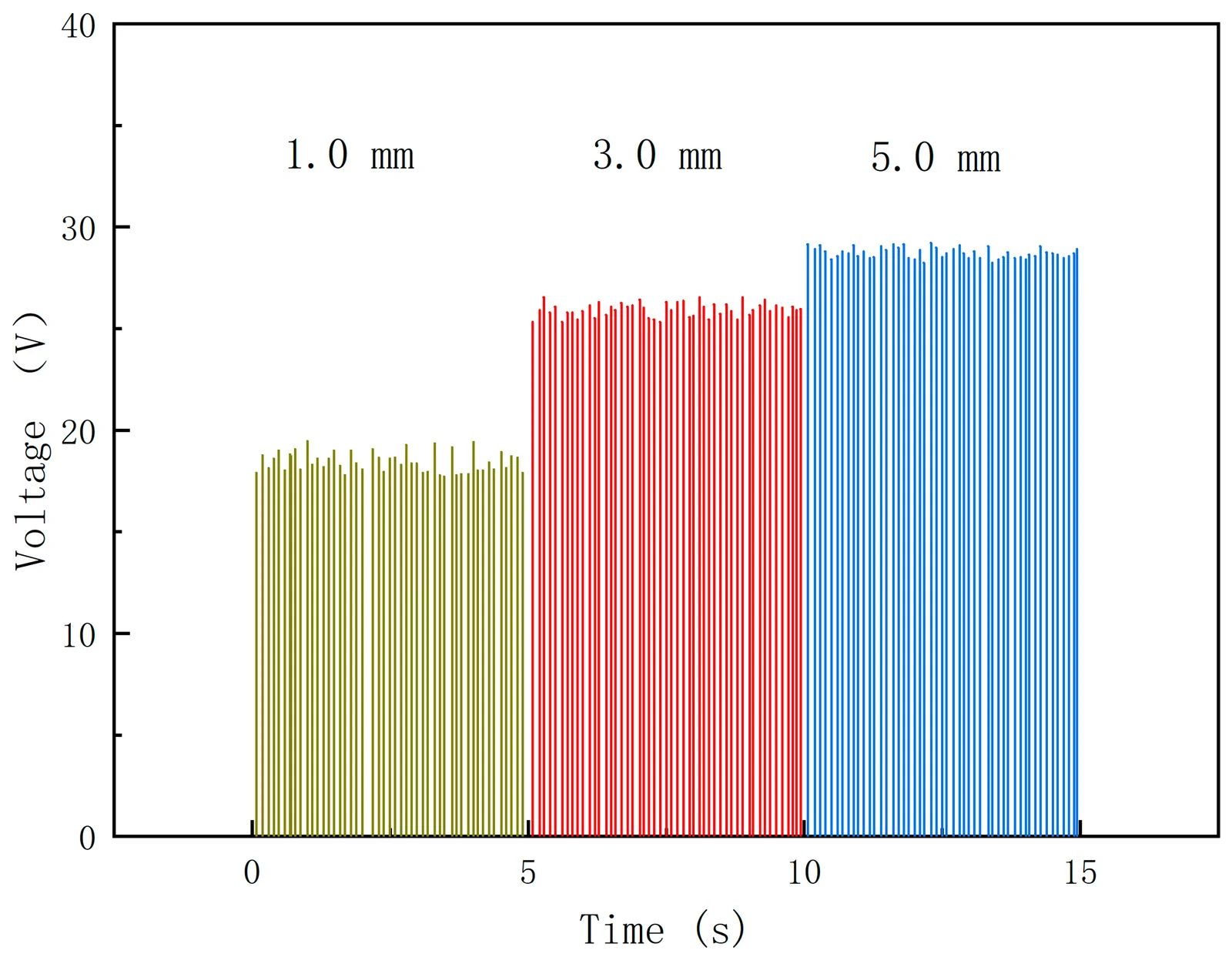
Correlation between film thickness and output performance.

To explore the effect of film thickness on output performance, three porous films (1.0 mm, 3.0 mm, and 5.0 mm) were fabricated. All samples possessed a fixed pore size of 63 μm and identical planar dimensions of 1 × 1 cm. Experimental measurements were subsequently carried out on these prepared films. The corresponding output performance results are shown in Figure 4. The curves are horizontally offset by 2.5 s for better visual clarity. The analytical results indicate that the output characteristics of the film exhibit an upward trend as the film thickness increases. Specifically, compared with the film thickness of 1.0 mm, the 3.0 mm film achieves a 30% improvement in electrical output performance. By contrast, although the 5.0 mm thick film presents an 11% performance improvement relative to the 3.0 mm counterpart, such an increment is far less remarkable than that obtained in the thickness range from 1.0 mm to 3.0 mm. In addition, the 1.0 mm thick film is prone to structural damage during packaging operation; by contrast, the 5.0 mm film exhibits excessive rigidity and poor wearing comfort. Careful consideration of packaging feasibility and wearable comfort indicates that the 3.0 mm film strikes an optimal balance, so it is chosen for subsequent experiments.

#### 3.1.3. Influence of Multi-Walled Carbon Nanotubes (MWCNTs) Doping Content on Output Performance

**Figure 5:**
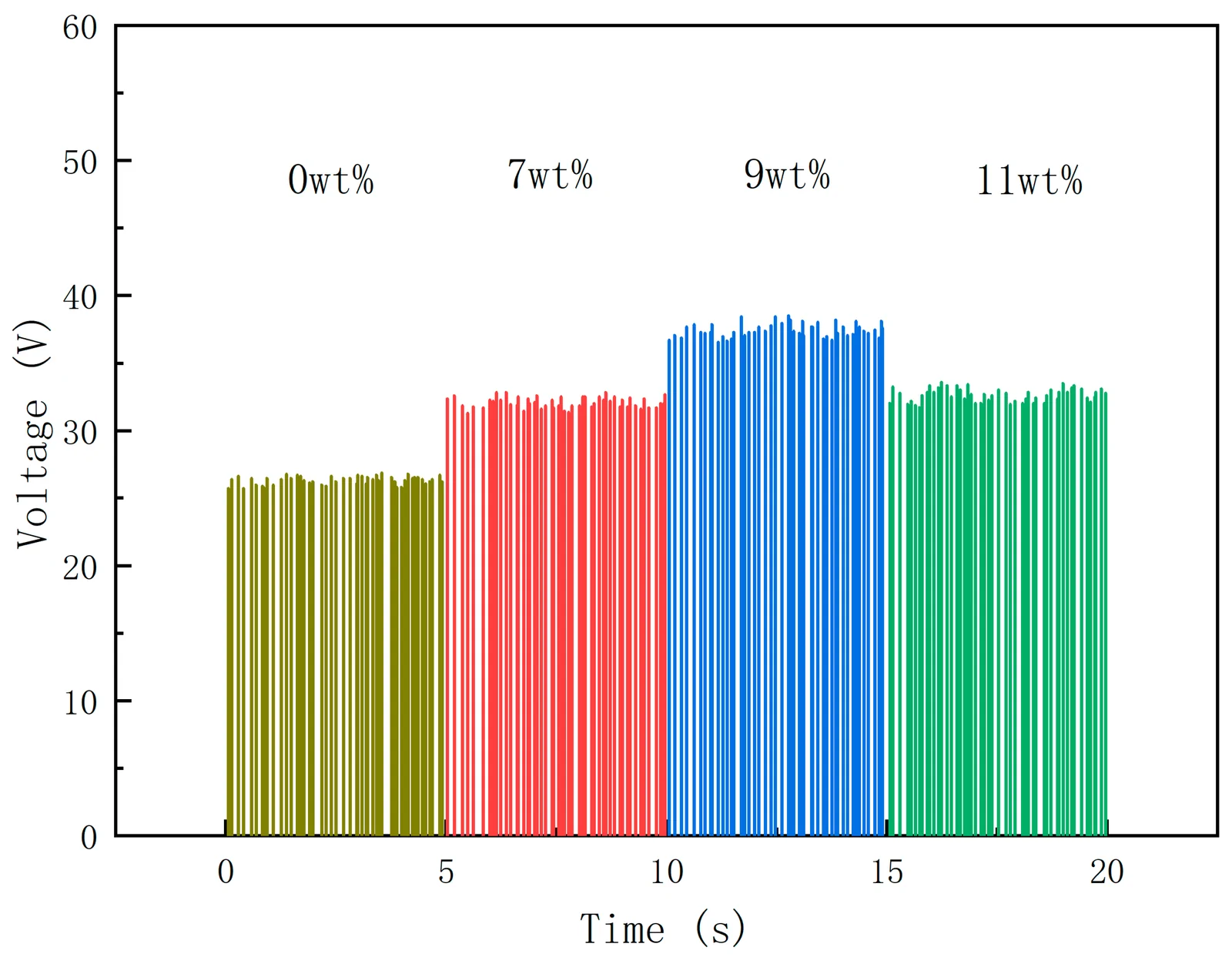
Influence of MWCNTs doping content on output performance.

Films with pore sizes of 63–75 μm, a thickness of 3.0 mm, and 0 wt% MWCNTs in the above tests were adopted as the fundamental control group. In total, four porous films (0 wt%, 7 wt%, 9 wt%, and 11 wt% MWCNTs) were prepared, and comparative tests were further carried out on the three MWCNT-doped films. The corresponding test results are illustrated in Figure 5. The curves are horizontally offset by 2.5 s for better visual clarity. The analytical results show that the output performance of the porous film with 7 wt% doping is increased by 19% compared with the undoped sample, and the performance of the porous film with 9 wt% doping is enhanced by 42%. By contrast, the performance improvement is only 22% for the 11 wt%-doped porous film. The doping of MWCNTs can effectively improve the output performance of porous films. However, the output performance presents a declining trend when the MWCNTs content exceeds a certain threshold. This phenomenon is attributed to the severe agglomeration of MWCNTs at high concentrations, which hinders their uniform and effective dispersion within the PDMS matrix. Consequently, the doping concentration of 9 wt% is selected as the optimal experimental parameter for subsequent investigations. It should be noted that this work focuses on the open-circuit voltage of the TENG. Other electrical performance parameters were not characterized in this experimental design, and comprehensive multi-parameter tests will be performed in future research.

#### 3.1.4. Flexibility and Fatigue Resistance Tests

**Figure 6:**
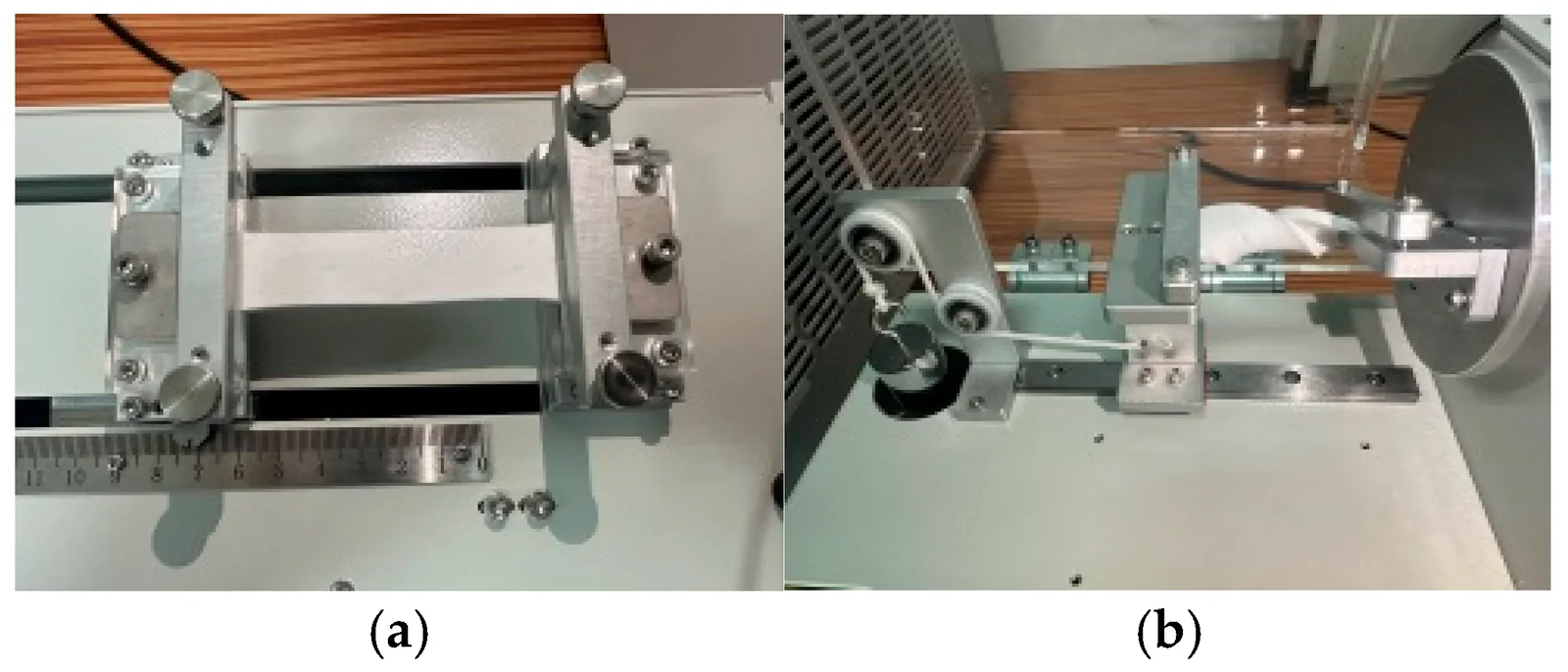
Flexibility and fatigue resistance tests. (**a**) 140% strain, 1 Hz tensile fatigue; (**b**) 180° cyclic torsion. Tests performed at 25 °C under ambient conditions.

The fatigue resistance and flexibility of the sensitive probe are also crucial evaluation indicators for the sensor. The flexibility and fatigue performance of the film were investigated by repeatedly applying tensile and torsional deformation under a certain applied stress. In this experiment, the PDMS film with a thickness of 3 mm and a pore size of 63 μm was selected for testing. Fatigue cycling tests were conducted under an applied strain of 140% and a constant cycling frequency of 1 Hz, and the device exhibited a peak open-circuit voltage of 6.1 V. When subjected to cyclic torsion at an angle of 180°, the maximum open-circuit voltage of the sensor reached 10.3 V. All tests were performed at room temperature (25 °C) under ambient atmospheric conditions. No obvious cracks appeared on the composite film after 3000 stretching and torsion cycles. The normalized output retention of the TENG-based flexible PDMS/MWCNTs composite sensor was evaluated over these 3000 cycles. The sensor maintained a relatively stable normalized output voltage upon repeated stretching and torsion deformation, verifying that the porous PDMS film possesses excellent flexibility and durability. Figure 6 shows photographs of the stretching and torsion test apparatus used for the fatigue measurement.

#### 3.1.5. Air Permeability Test

**Figure 7:**
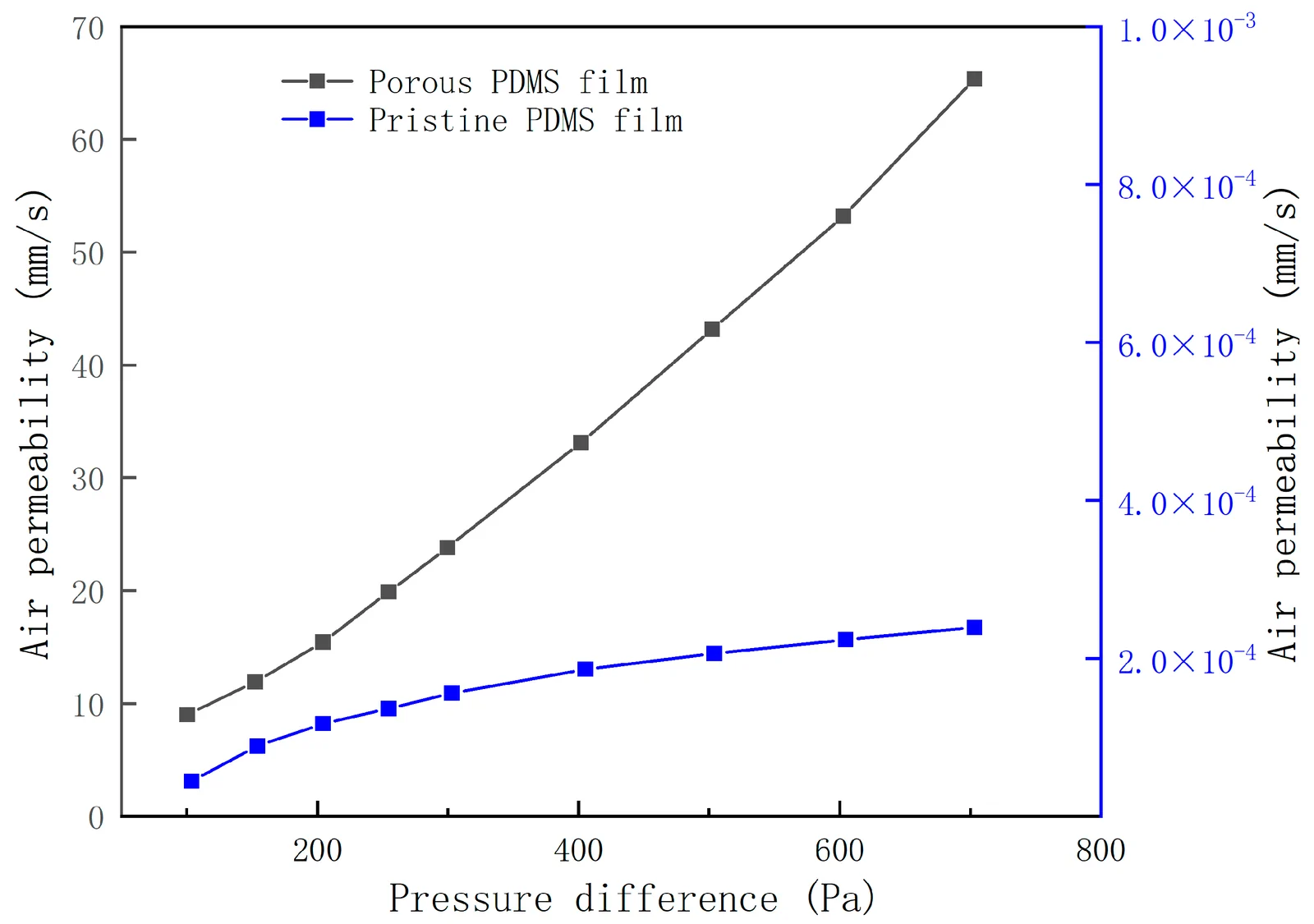
Air permeability of porous PDMS film and pristine PDMS film.

The air permeability of the porous PDMS film and pristine PDMS film was characterized to evaluate the wearing comfort of flexible finger-pressure sensors. The porous PDMS film developed in this work is intended for flexible finger pressure sensing. To ensure intimate and comfortable contact with human skin, excellent air permeability of the film is essential. Accordingly, the air permeability of both a 3 mm thick porous PDMS film, fabricated using refined sugar particles with a diameter of 63 μm as the sacrificial template, and a dense pristine PDMS film was evaluated. Owing to the substantial difference in air permeability between the two samples, distinct instruments were employed for characterization: the VAC-V2 gas permeability tester was used to measure the dense pristine PDMS film, and the air permeability of all specimens was subsequently calculated. As shown in Figure 7, the porous PDMS film exhibited a significantly higher air permeability than its dense counterpart, demonstrating that the porous structure effectively enhances the air permeability of PDMS films and is favorable for wearable sensing applications.

### 3.2. Characterization and Analysis of Composite Films

#### 3.2.1. Morphology Characterization of Sugar Particles

**Figure 8:**
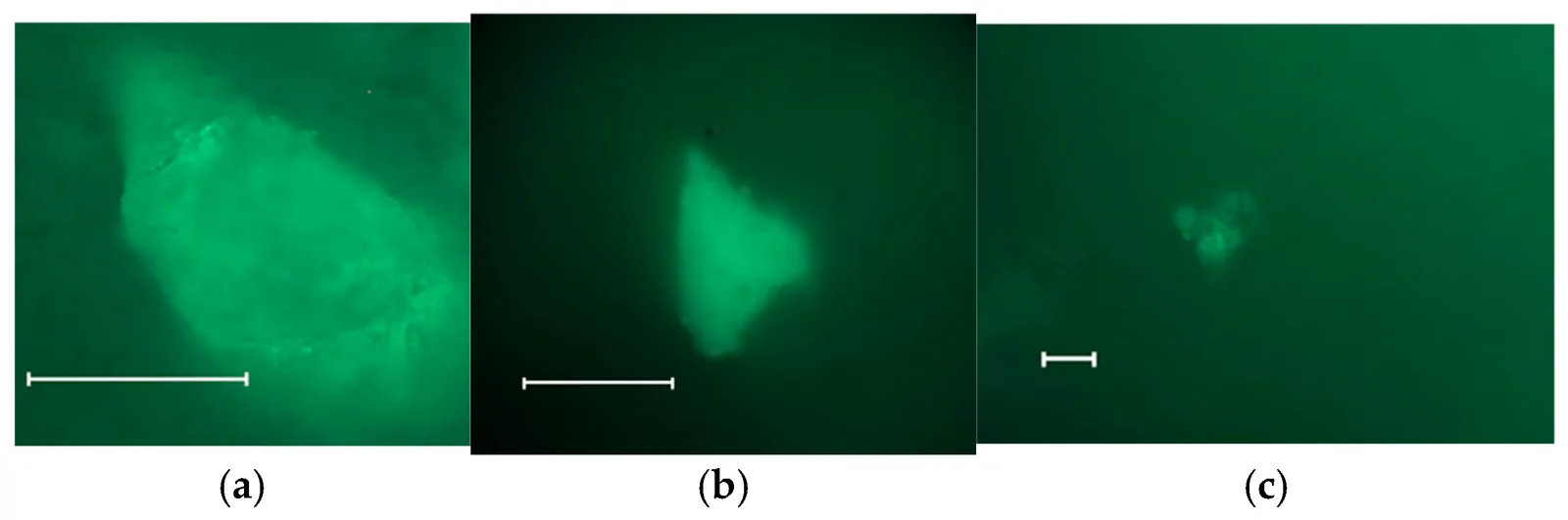
A 5× objective was employed for (**a**), and a 10× objective was employed for (**b**,**c**).

In this work, three types of sugar particles with different sizes were systematically characterized for the fabrication of porous PDMS films. Three sets of sieves with mesh sizes of 30/35 mesh, 80/100 mesh, and 200/250 mesh were employed, where mesh denotes the number of openings per linear inch. The corresponding particle size ranges for (a), (b), and (c) are 500–600 μm, 150–200 μm, and 63–75 μm, respectively. To accurately characterize the particle size range of each group, a sampling method was adopted. Dry sucrose granules were loosely dispersed onto glass slides and cover slips. A 5× objective was employed for imaging large particles (300–600 μm), whereas a 10× objective was used for smaller fractions (63–300 μm). Particle surface morphology was imaged at appropriate focal planes under a fluorescence optical microscope, as shown in Figure 8.

#### 3.2.2. Internal Structure Characterization of Porous Structures with Different Sizes

**Figure 9:**
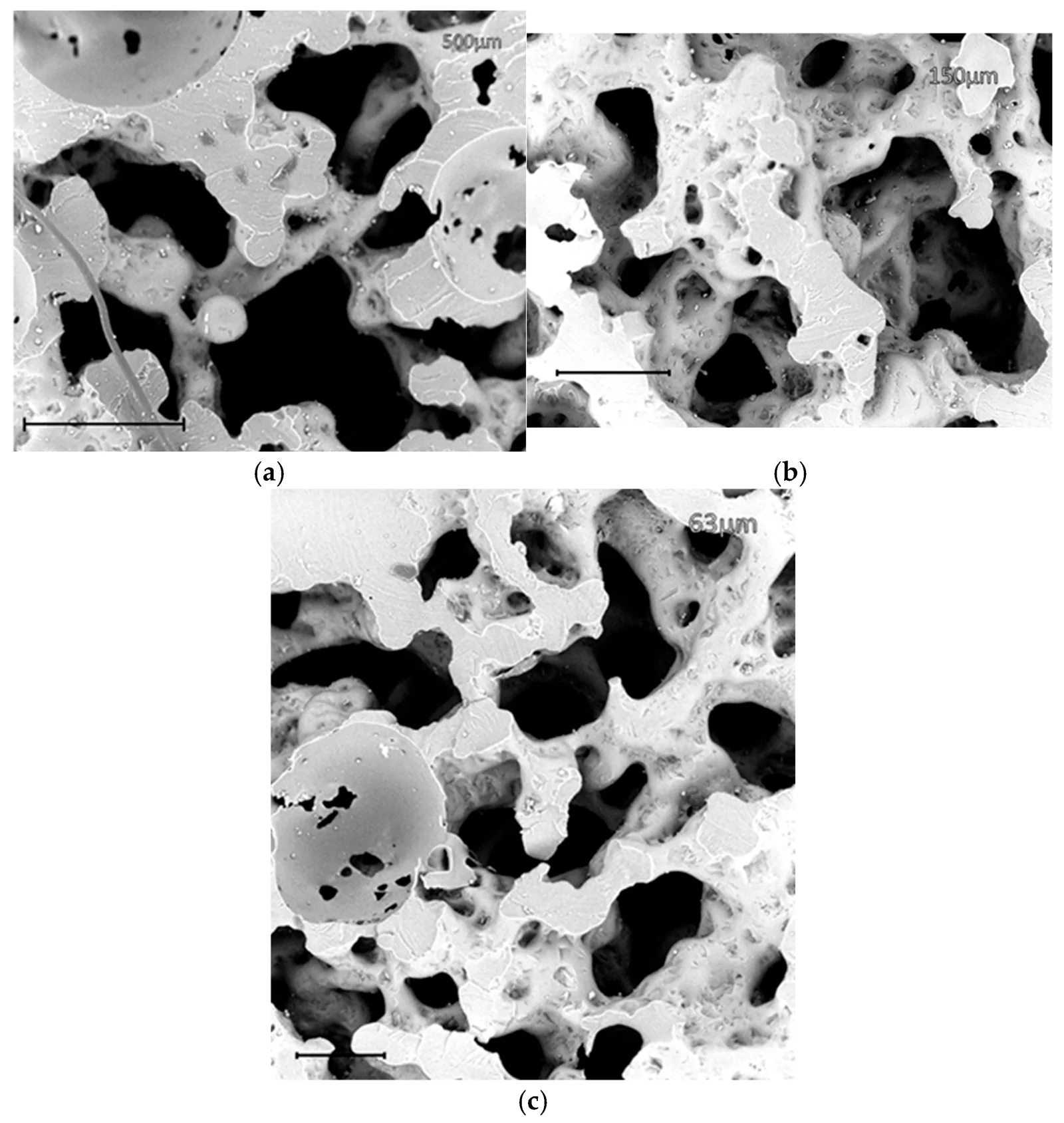
SEM micrograph of the composite sample with 0 wt% MWCNTs. (**a**) Sample with filler particle size of 500 μm; (**b**) Sample with filler particle size of 150 μm; (**c**) Sample with filler particle size of 63 μm. Images were acquired at an accelerating voltage of 5 kV and a working distance of 8.5 mm.

In the experiment, refined sugar particles of different sizes were used to fabricate porous PDMS films with 0 wt% MWCNTs, which possessed varied internal pore sizes. To evaluate the effect of particle size on the final pore structure, all films were cut into identical dimensions and subjected to dust removal treatment. The tests were carried out under an accelerating voltage of 5 kV with a working distance of 8.5 mm for image acquisition. Different magnifications were selected to observe the pore structure and filler distribution of the samples. Since PDMS-based composite films are dielectric materials, all specimens were subjected to gold sputter coating prior to SEM observation to eliminate surface charge accumulation. The corresponding results are presented in Figure 9. It was observed that the porous PDMS film templated by 500 μm sugar particles possessed the largest pore size, while the sample prepared using 150 μm particles exhibited a moderate pore size, and the film derived from 63 μm particles showed the smallest pore dimension. This indicates that the resultant pore size decreases gradually with the reduction in sacrificial particle size. Comparative analysis of the SEM images clearly reveals the significant influence of sugar particle size on the internal microstructure of PDMS films. This conclusion provides a valuable reference for subsequent related research.

#### 3.2.3. Characterization of PDMS/MWCNTs Composite Porous Structures

**Figure 10:**
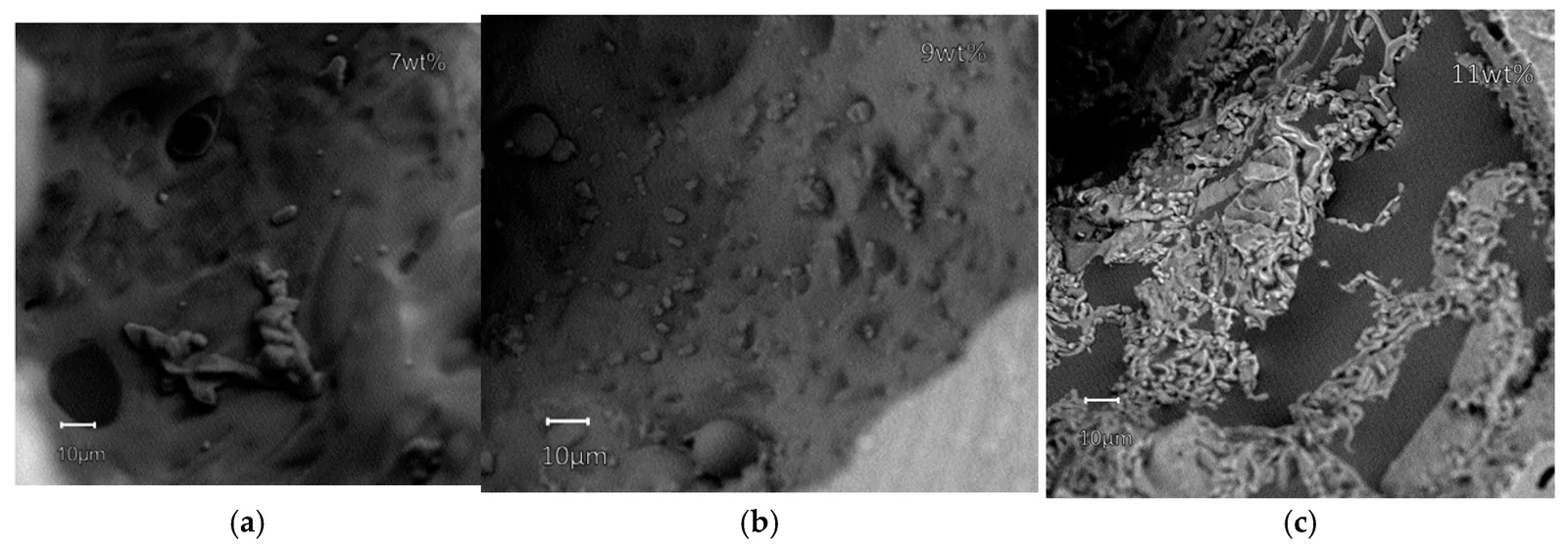
SEM micrograph of the composite sample. (**a**) 7 wt% doping content; (**b**) 9 wt% doping content; (**c**) 11 wt% doping content. The sample was mounted with conductive carbon tape and sputter-coated with an 8–12 nm Pt-Pd layer to suppress charging artifacts.

In this experiment, three different mass fractions of MWCNTs, namely 7 wt%, 9 wt%, and 11 wt%, were selected for doping to enhance the film performance. The corresponding SEM images are presented in Figure 10. The samples were adhered to the specimen stub using conductive carbon adhesive tape to ensure electrical contact. Subsequently, an 8–12 nm Pt-Pd layer was sputter-deposited on the sample surface to mitigate charging artifacts during SEM imaging. The analysis reveals that at 7 wt% MWCNT doping, the distribution is relatively sparse. In contrast, a relatively uniform dispersion is achieved at a doping content of 9 wt%. However, when the doping content increases to 11 wt%, severe agglomeration of MWCNTs occurs, preventing their uniform dispersion within the film matrix. Agglomeration leads to the degradation of electrical output performance. It should be noted that the low-magnification SEM images cannot clearly distinguish individual MWCNTs within the composite matrix. Higher magnification is required to resolve the morphology of isolated MWCNTs. Therefore, above 9 wt% MWCNT loading, filler-induced conductive pathway formation and charge leakage serve as plausible competing mechanisms responsible for the degraded electrical output. Nevertheless, this analysis remains qualitative. Further quantitative characterization of conductivity, resistivity, and dielectric loss is required in future work to quantitatively decouple dielectric enhancement, filler agglomeration, and charge leakage effects at high MWCNT fractions [7]. 

### 3.3. Sensitivity Characterization

**Figure 11:**
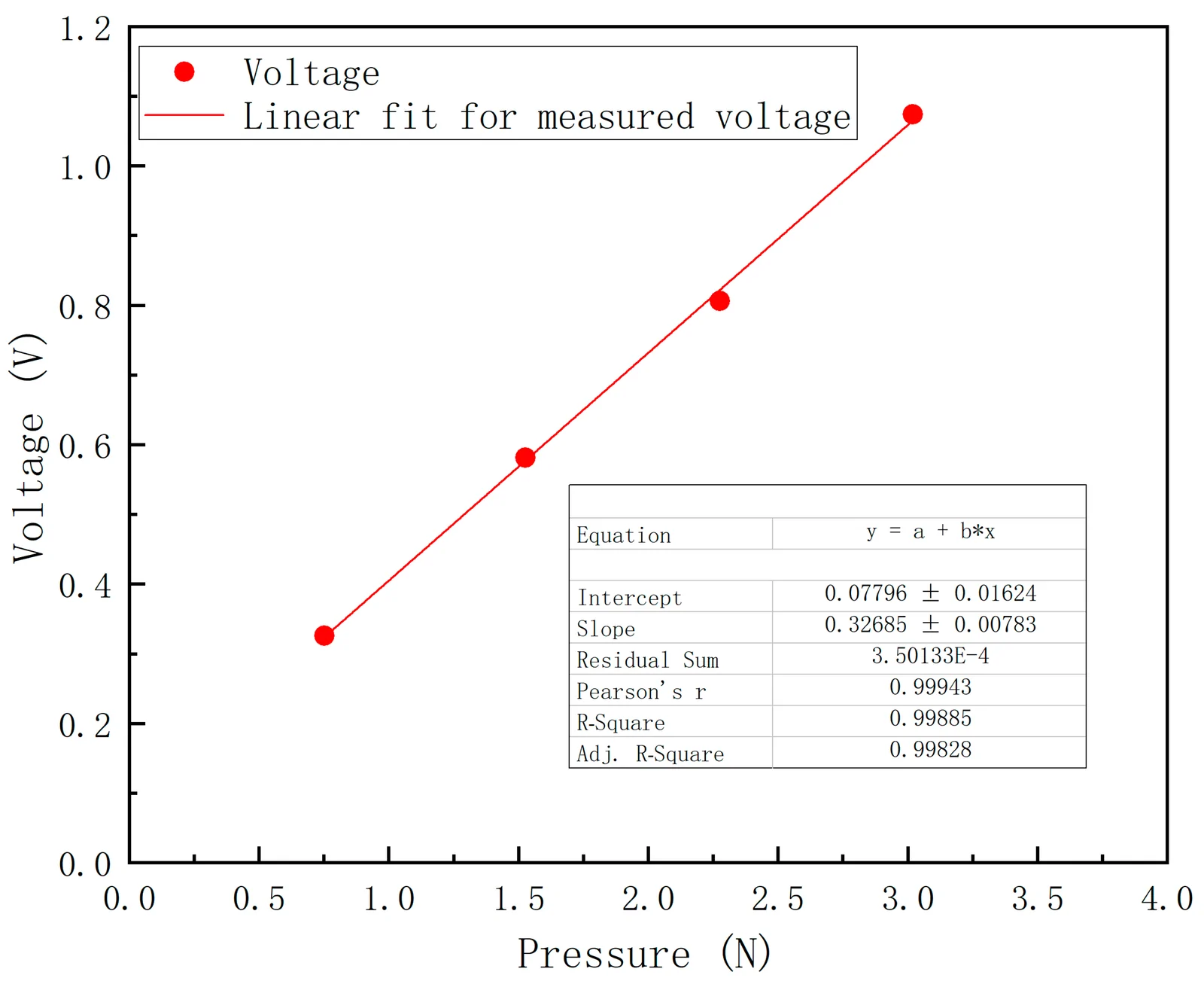
Linear fit for measured voltage. Output voltage was measured under applied forces of 0.75 N, 1.5 N, 2.25 N and 3.0 N; 250 data points were acquired for each condition.

Sensitivity characterization was performed by quantifying output-voltage variations under applied forces of 0.75 N, 1.5 N, 2.25 N, and 3.0 N. Under each condition, 250 data points were collected, and the peak output voltage served as the reference for sensitivity calculation. The data were fitted in Origin, as shown in Figure 11, yielding a force-based sensitivity of 0.328 V/N (0.0328 V/kPa). As listed in Table 1, the MWCNT/PDMS triboelectric sensor offers distinct advantages: low-cost fabrication from readily available materials (MWCNT/PDMS film, double-sided Kapton, and sugar) without complex etching, carbonization, or liquid metal encapsulation; a working range of 7.5–30 kPa matching typical finger-pressing pressure; and stable performance over 3000 cycles, demonstrating competitive durability. Nevertheless, limited by experimental conditions, the maximum measurable pressure is 30 kPa, making the device more suitable for low-pressure human–machine interaction than for high-pressure applications.

### 3.4. Pulse Data Acquisition

Healthy young student volunteers aged 18–30 years were recruited for the test. All participants had no irregular lifestyles, such as recently staying up late, and avoided strenuous exercise before measurement so as not to interfere with the normal rhythm and frequency of the arterial pulse. A total of four qualified college students, including two males and two females, were finally selected for the repeatability test. Prior to data collection, each participant was required to sit quietly for five minutes to relax physically and mentally and regulate their breathing, ensuring the smooth implementation of the experiment. During the measurement, all participants maintained an upright sitting posture with the left arm placed flat and relaxed on the table in a palm-up position. The arm was adjusted to approximately the same horizontal level as the heart, and both the body and arm were kept stable; the sensor finger sleeve was worn properly and gently pressed against the tester’s radial artery. Participants were asked to breathe naturally throughout the test and minimize body and arm movement to avoid interference with signal acquisition. Each volunteer was measured for 60 s under a constant applied force. After each test, the sensor was removed, and a five-minute resting interval was arranged before data recording and the next measurement. This procedure was repeated five times for each participant, and the pulse data from each test were recorded separately. After completing five repeated measurements for the first participant, the same test protocol was performed on the next volunteer in sequence.

**Figure 12:**
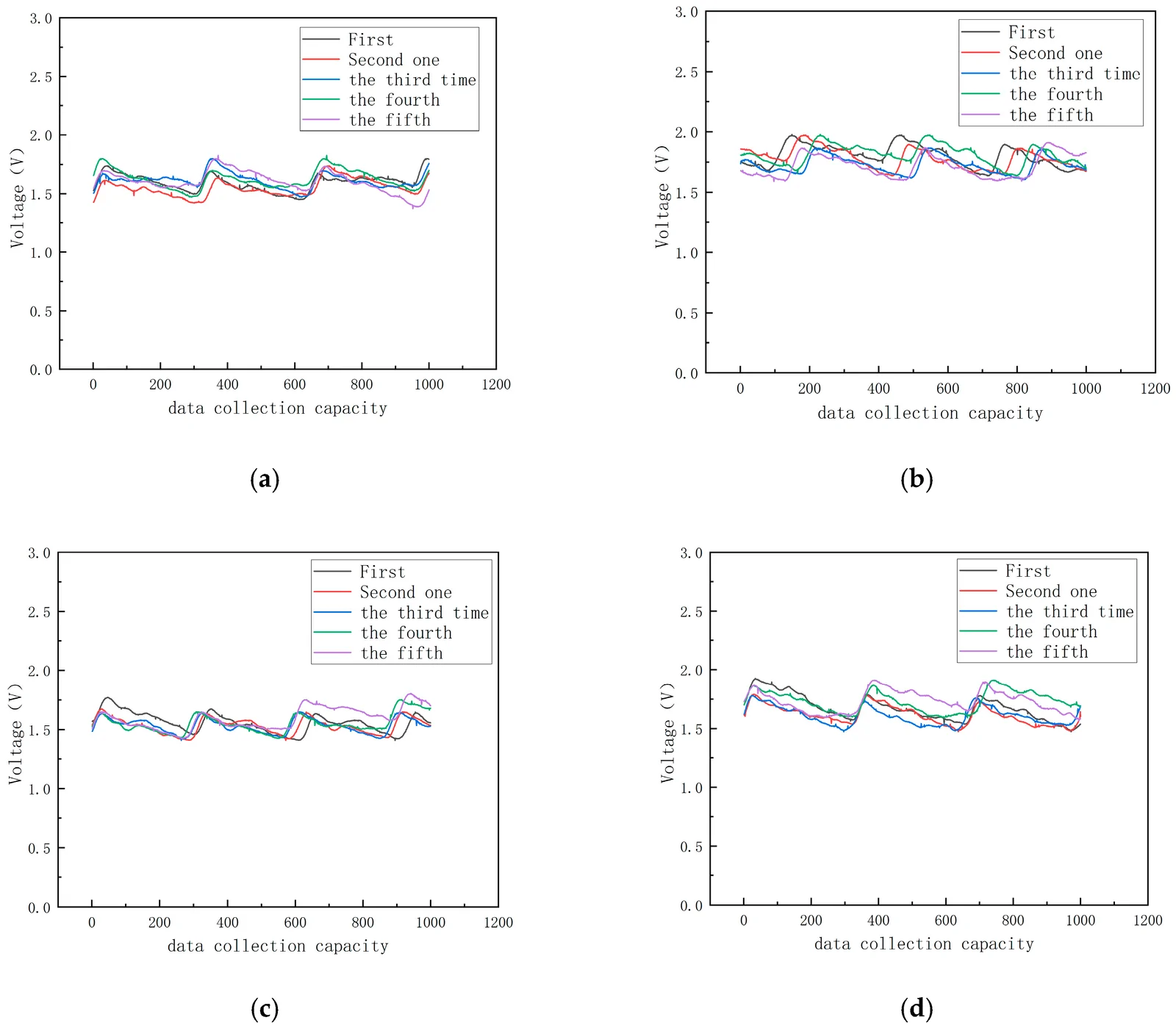
Comparative tests of five-time data from four subjects using a pulse-monitoring instrument. (**a**) Student 1; (**b**) Student 2; (**c**) Student 3; (**d**) Student 4. upright sitting, left palm-up arm at heart level, sensor on radial artery. 60 s recording per subject under constant force; 5 min rest between tests.

**Figure 13:**
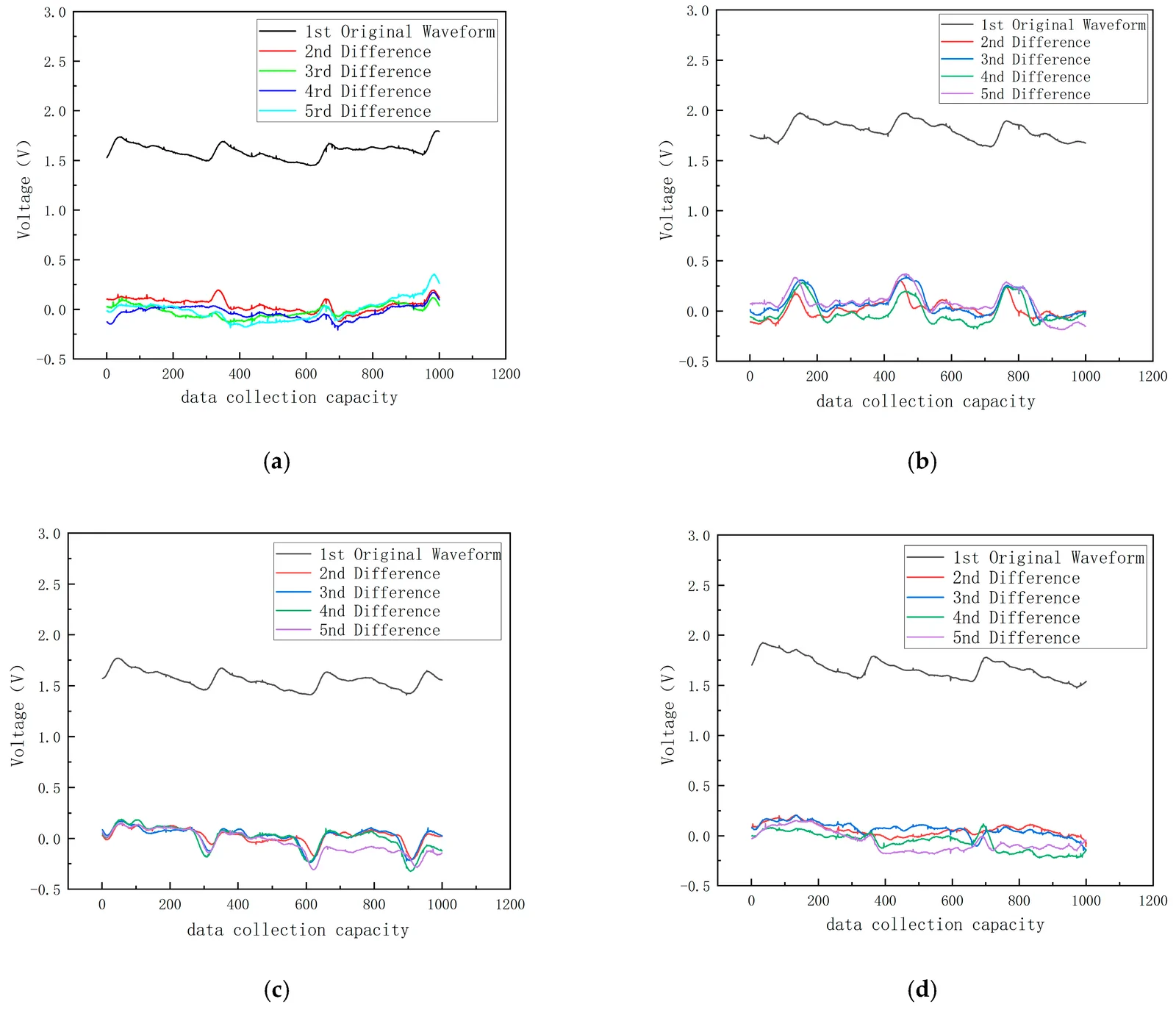
Comparative analysis of differential test signals based on the first-trial data as the reference. (**a**) Student 1; (**b**) Student 2; (**c**) Student 3; (**d**) Student 4.

Random segments were extracted from five sets of pulse data for each participant for comparative analysis, and the corresponding results are presented in Figure 12. It can be observed that the pulse waveforms acquired from five repeated measurements of the same subject exhibit a high degree of consistency with only minor fluctuations. Such slight fluctuations mainly originate from random interference induced by tiny variations in finger pressing force during pulse detection, demonstrating the excellent operational stability of the sensor in repeatability tests. All pulse waveforms were aligned based on the peak position of each pulse cycle. To quantitatively evaluate the repeatability of the sensor, the first recorded pulse waveform of each participant was selected as the reference baseline. The voltage deviations between the baseline and the subsequent four repeated measurements were calculated, and the maximum voltage deviation was determined to be 0.38 V. The distribution of signal deviations across repeated tests is presented in Figure 13. The analytical results indicate that slight deviations among multiple measurements are mainly attributed to respiratory movement of the subject, tiny arm displacement, and subtle changes in finger pressing state during testing. From the overall data distribution, most deviation values remain at a low level, with a maximum deviation of no more than 0.38 V, except for a few discrete outliers caused by transient interference. Comprehensive analysis confirms that the developed sensor possesses satisfactory signal consistency and stability in repeated measurements, which fully meets the repeatability requirements for practical pulse signal acquisition.

## 4. Conclusions

The sensor fabricated by the sugar sacrificial template method in this work exhibits distinct practical advantages. First, sugar granules are low-cost and easily accessible, avoiding expensive microspheres and hazardous chemical pore-forming agents. Second, the one-step fabrication process simplifies manufacturing procedures and reduces equipment requirements. Furthermore, the porous structure simultaneously improves the mechanical flexibility and air permeability of the composite film, enabling long-term comfortable skin contact for wearable pulse monitoring. Optimal parameters were determined via systematic tests and SEM morphological characterization: a particle size of 63 μm, a film thickness of 3.0 mm, and an optimal multi-walled carbon nanotube (MWCNT) loading of 9 wt%. Subsequently, the TENG-based flexible PDMS/MWCNTs composite sensor was integrated with the custom-designed signal-conditioning circuit, enabling real-time visualization of pulse signals via the upper computer system. Five repeated pulse measurements were performed on four volunteers for comparative data analysis. The results demonstrate high consistency among the pulse waveforms obtained from five repeated tests for each subject. Minor discrepancies mainly arise from respiratory motion and slight external disturbances. The overall signal deviation is limited, with the maximum voltage difference being within 0.38 V. In conclusion, this fabrication strategy offers a feasible route for scalable, low-cost self-powered physiological sensing devices and possesses broad application prospects in healthcare monitoring and wearable physiological sensing.

## Figures and Tables

**Table 1:** Performance comparison of various pressure sensors.

Fabrication Materials	Sensing Mechanism	Measurement Range	Sensitivity	Cycle Number	Reference
PET, graphene, acrylic and PDMS	Triboelectric sensor	10.6–101.7 kPa	0.274 V/kPa	2000	[9]
MXene/Cotton fabric, PDMS and PI	Piezoresistive sensor	0–1.30 kPa 1.30–10.25 kPa 10.25–40.73 kPa 40.73–160 kPa	5.30 kPa^−1^ 2.27 kPa^−1^ 0.57 kPa^−1^ 0.08 kPa^−1^	>1000	[10]
TPU/PVDF-HFP nanofiber, liquid metal, Al and PTFE	Capacitive sensor	90–400 kPa	0.071 V/kPa	1000	[11]
MWCNT/PDMS, double-sided Kapton and sugar	Triboelectric sensor	7.5–30 kPa	0.0328 V/kPa	3000	This work

## Data Availability

The raw data supporting the conclusions of this article will be made available by the authors on request.

## Abbreviations

ADC	Analog-to-Digital Converter
BPPW	Blood pressure pulse waveform
CAM	Citric Acid Monohydrate
CNT	Carbon Nanotube
FPCB	Flexible Printed Circuit Board
FTENG	all-fibrous superhydrophobic triboelectric nanogenerator
F-TENG	Fabric-based integrated TENG
H-NG	Hybrid Nanogenerator
LL-VNS	Low-level vagus nerve stimulation
MWCNTs	Multi-Walled Carbon Nanotubes
MXene	Two-dimensional transition-metal carbide/nitrid
MP-TENG	MXene/poly(methyl methacrylate) triboelectric nanogenerator
PDMS	Polydimethylsiloxane
PTFE	Polytetrafluoroethylene
PVA	Polyvinyl Alcohol
SEM	Scanning Electron Microscopy
TENG	Triboelectric Nanogenerator
USART	Universal Synchronous/Asynchronous Receiver/Transmitter
3DFIFTENG	3D double-sided interlocked fabric triboelectric nanogenerator
